# Green and Pleasant Lands: The Affective and Cerebral Hemodynamic Effects of Presence in Virtual Environments During Exercise

**DOI:** 10.1177/00315125221146614

**Published:** 2022-12-21

**Authors:** Leighton Jones, Jonathan Wheat

**Affiliations:** College of Health, Wellbeing and Life Sciences, 7314 Sheffield Hallam University, UK

**Keywords:** hedonic exercise, near infrared spectroscopy, virtual reality, distraction during exercise, exercise adherence

## Abstract

Pleasant exercise experiences increase the likelihood of exercise adherence, and innovative strategies to promote consistently pleasant exercise experiences are needed. In this study we compared a novel nature-based virtual reality environment, a nature-based 360° video, and a control condition to test the hypothesis that greater presence in virtual space would promote positive affective experiences during exercise. Moreover, we assessed prefrontal cerebral hemodynamics using near infrared spectroscopy to explore possible neural underpinnings of dissociative strategies during exercise. Twelve participants (*M* = 26.2, *SD* = 7.7 years; *M* BMI = 25.5, *SD* = 5.2 kg/m^2^) completed a maximal aerobic test and three exercise conditions (Control, Virtual Reality [VR], and 360° video). The two experimental conditions differed in terms of the participants’ sense of presence (VR eliciting greatest presence), and all conditions utilized similar exercise intensity. The VR condition setting was a virtual mountain forest trail, and the 360° video was of a forest road. The 360° video was perceived as the most distracting (*p* = .023, *d* = 1.07), pleasant (*p* = .007, *d* = .75), and enjoyable (*p* = .029; *d* = .82) condition. ΔHbDiff data indicated that the control condition caused the greatest prefrontal brain activation (*p* = .008, *d* = .84). Presence was not a salient factor in distracting participants from bodily sensations during exercise, but immersion in a stimulus was. These results provide support for using head-mounted displays during exercise as a strategy to increase pleasure, with practical implications for practitioners, researchers, and individuals.

## Introduction

The persistent prevalence of physical inactivity demands innovative strategies to engage insufficiently active individuals. Physical activity (PA) is a broad term capturing human bodily movement, and global and national guidelines have been developed that recommend suitable amounts of PA to promote its physical and mental health benefits (Bull et al., 2020). These guidelines include moderate and vigorous intensity aerobic activity, and an effective way to meet PA recommendations is through structured exercise. Yet, it is well known that exercise adherence is often poor (Robison & Rogers, 1994). While attempts to improve rates of exercise initiation and adherence have led to the design of varied strategies, these have largely been unsuccessful (Hallal et al., 2012), raising a need for alternative theoretical approaches (Ekkekakis, 2017). Many existing strategies for promoting exercise adopted a cognitivist approach predicated on a rational-education model (Weare, 2002). Recently, new models have proposed a different approach to help researchers contextualise and inform their efforts to engage inactive individuals in exercise.

There has been an increasing acknowledgement of the roles that Type 1 (automatic) and Type 2 (reflective) decision-making processes play in determining exercise behavior. The Affective-Reflective Theory (ART) of physical inactivity and exercise (Brand & Ekkekakis, 2018) recently proposed that reflective evaluations depend upon automatic affective valuations (assignment of pleasant or unpleasant valence) to exercise-related stimuli and the availability of self-control resources. Ultimately, the interaction of automatic and reflective processes determines whether an individual will or will not continue to engage in exercise behavior. The proposed salience of automatic affective responses (Type 1 process) in influencing other cognitions (Type 2 process) suggests that affective responses play a particularly important role in influencing exercise behavior. Therefore, better understanding of how to improve affective responses to exercise is a worthwhile direction for new research. Consistently pleasant exercise experiences can help to create positive automatic associations with exercise that increase the likelihood of future engagement in it. Rhodes and Kates (2015) identified that in-task (vs. post-task) affective responses are significant mediators of future exercise behavior and that additional research exploring strategies to facilitate them is warranted.

Pleasure during exercise can be influenced by intrinsic components of exercise that include frequency, intensity, time, and type (FITT) and by extrinsic components that include environmental manipulations (e.g., listening to music during exercise) outside of the FITT principles (see Jones & Zenko, 2021). Surprisingly, few extrinsic strategies have been developed to increase pleasure during exercise. Previous work on in-task extrinsic strategies has shown a positive relationship between exercise participants’ mental dissociation from unpleasant stimuli and their pleasure during moderate-to-heavy intensity exercise (e.g., distracting music-videos, Jones et al., 2014). On evidence that audio-visual stimuli can serve as effective distractors during exercise, Bourke et al., 2021 stated that “virtual reality may have the potential to increase pleasurable feelings during moderate-to-vigorous physical activity” (p. 175). Recent attempts to examine the role of emerging technologies such as consumer-level head mounted displays have shown some efficacy in positively enhancing pleasure during exercise. Bird et al., (2020) found that 6-minute bouts of cycling while experiencing a virtual cycling game promoted a pleasant exercise experience compared to a control condition. However, Calogiuri et al., (2018) compared a 10-minute nature walk with a treadmill walk while watching a 360° video of the same nature walk and found that (a) participants reported greater pleasure and enjoyment during the nature walk; and (b) qualitative feedback raised concerns about cybersickness during the 360° video. Thus, efforts to reduce cybersickness should feature prominently in future exercise designs if such strategies are to be effective at promoting pleasure. Using images and video footage of nature during exercise has been explored in several other studies, with some positive effects on emotional responses (e.g., Jones et al., 2014; Yeh et al., 2017). The use of natural environments as a pleasant visual stimulus during exercise holds promise for positively improving the exercise experience compared to indoor exercise that includes no purposefully selected visual stimuli. An alternative approach to virtual games and to real versus virtual nature exposure was examined by Jones and Ekkekakis (2019) who sought to immerse overweight participants in an emotionally positive audio-visual stimulus (music-videos delivered via a head-mounted display) with the aim of promoting greater mental dissociation from unpleasant physical sensations during exercise. They compared this immersion to more traditionally delivered stimuli (music-videos delivered on television screens) and found that stimulus immersion in a head-mounted display was associated with greater pleasure and enjoyment. In addition to stimulus immersion, “presence” has been cited as a relevant factor in stimulus delivered via head-mounted displays (Slater, 2018).

Immersion concerns the technological aspects of virtual environment (e.g., field of view, display size, resolution, and frame rate), and it refers to the extent to which the user is “shut-off from the real world by a VR system” (Weech et al., 2019, p. 3). However, high immersion does not ensure presence. Presence has been described as the illusion of *being there* (Heeter, 1992), and it is broadly considered the primary goal to achieve a successful VR experience (Weech et al., 2019). To foster a sense of presence in the VR environment, the environment should facilitate motor affordances, have a degree of interaction with the environment, and have high fidelity and realism (see Slater, 2018). To date, there have been no studies that have sought to experimentally test the role of presence in VR stimulus during exercise. Further examining the role of presence in determining pleasure during exercise could help guide developers and researchers and provide further strategies for exercise practitioners to consider when promoting pleasurable exercise experiences.

Among a limited number of strategies that have shown a capacity to promote a pleasurable and enjoyable exercise experience (e.g., music [Jones et al., 2017], music and video [Hutchinson et al., 2015]; virtual reality [Bird et al., 2020], and imagery [Tempest & Parfitt, 2013]), neural mechanisms underpinning the efficacy of these approaches have received scant attention. Preliminary attempts to understand the cerebral mechanisms underpinning these strategies have included the use of near-infrared spectroscopy (fNIRS) and have provided support for specific theorized mechanisms (cf. Karageorghis et al., 2017). Tempest et al., (2014) showed that, at intensities above ventilatory threshold (T_vent_), cerebral activation of the dorsal regions of the prefrontal cortex increased (as measured by differences in cerebral oxygenation). Their findings demonstrated an inverse relationship between affective responses and cerebral activation, with pleasure declining as exercise intensity and prefrontal cerebral activation increased. In a study of right dorsal lateral prefrontal cortex (dlPFC) activity during “heavy” exercise under immersive stimuli, Jones and Ekkekakis (2019) found that overweight individuals with a preference for low intensity exercise reported less pleasure and exhibited significantly higher levels of right dlPFC activation during a control cycling condition compared to exercise with an audio-visual stimulus. They concluded that lower levels of pleasure during exercise appear to be coupled with greater right dlPFC activation that may indicate the participants’ efforts to cognitively regulate their unpleasant experience. Further, the audiovisual stimuli in Jones and Ekkekakis (2019) attenuated the increased right dlPFC activity, indicating a reduced reliance on prefrontal affect regulation processes when distracted by the stimuli. These data provide preliminary insights into neural mechanisms underpinning the efficacy of immersive strategies during exercise, but additional research is needed.

The intensity of exercise is an important intrinsic determinant of the affective response (Jones & Zenko, 2021). The Dual-Mode Model (Ekkekakis, 2003) posited that low-intensity exercise typically leads to positive affective responses (i.e., increased pleasure), while heavy exercise intensities (between T_vent_ and Respiratory compensation point [RCP]) elicit variable affective responses, and severe exercise intensities (above RCP) result in a sharp decline in pleasure. Variable responses during heavy exercise are a consequence of competing interoceptive (e.g., blood lactate) and cognitive factors (e.g., self-efficacy), with a shift towards a more predominant role for interoceptive factors as intensity increases. Heavy exercise is associated with greater physiological benefits compared to lower-intensity exercise (Foulds et al., 2014). Therefore, exercise intensities proximal to T_vent_ are appropriate to examine experimentally with strategies that may promote a more consistently pleasant exercise experience (owing to the variable response), and these intensities are meaningful for physiological benefits.

Our purposes in this study were to examine whether greater participant perceived presence in the virtual environment would influence the participants’ affective responses during exercise and to explore possible neural mechanisms underpinning these responses. We hypothesised that a virtual environment creating a stronger sense of presence would lead to the participants’ most positive affective responses and greater mental dissociations from unpleasant sensations. We also expected that activation of dlPFC would be highest in the absence of virtual stimuli during exercise.

## Methods

### Participants

Twelve participants (4 women, 8 men) between the ages of 19–47 years (*M* = 26.2, *SD =* 7.7 years) who were borderline overweight (*M* BMI = 25.5, *SD* = 5.2 kg/m^2^) and of average aerobic fitness (Peak $\dot{V}O_{2}$ *M* = 37.4, *SD* = 6.4 mL/kg/min) completed all exercise conditions. We did not collect information regarding these participants’ socioeconomic status. This small sample size was a consequence of resource constraints (Lakens, 2022) and COVID-19 pandemic restrictions. All study procedures were reviewed and approved by the institutional ethics committee at Sheffield Hallam University, UK. All participants provided written informed consent prior to participation.

### Measures

#### Manipulation Checks

##### Presence Questionnaire (PQ; Witmer & Singer, 1998)

We measured the participants’ sense of presence in the virtual environment with the PQ (omitting the 19 items relating to haptic and auditory subscales). PQ responses are provided on a 7-point Likert scale, with verbal anchors relevant to the individual questions (e.g., “How natural did your interactions with the environment seem?” followed by “Extremely artificial” to “Completely natural”). Internal consistency (Cronbach’s α) of this measure was .89 and .92 for the experimental conditions.

##### Simulator Sickness Questionnaire (SSQ; Kennedy et al., 1993)

We administered the SSQ to assess cybersickness. The SSQ comprises 16 items with the common stem statement “Circle how much each symptom below is affecting you right now” and descriptors such as “sweating”, and “nausea” are included among the list of symptoms. Responses are on a scale from “none” to “severe” with a weighted scoring system for three subscales of nausea, oculomotor, and disorientation creating a total score. Caserman et al. (2021) suggested that a total score over 40 indicated a “bad” intervention (i.e., problematic cybersickness). Cronbach’s α of the SSQ was .72 for the VR condition, .57 for the 360° video condition, and .84 for the control condition. These data reveal poor internal consistency of the measure for the 360° video condition.

### Exercise Intensity (% HRpeak)

Heart rate was recorded (Polar H10) throughout each condition and reported as %HRpeak. Peak heart rate was determined based on highest heart rate recorded during the maximal test.

#### Outcome Measures

##### Feeling Scale (FS; Hardy & Rejeski, 1989)

The FS is designed to measure affective valence (pleasure–displeasure); it is a single item measure with responses on an 11-point scale, ranging from +5 (“very good”) to −5 (“very bad”). The scale has previously demonstrated satisfactory validity (Hardy & Rejeski, 1989).

##### Attentional Focus (Tammen, 1996)

This single-item attention scale was administered to assess attentional focus (association–dissociation). Responses range from 0 (total associative focus) to 100 (total dissociative focus) and the participant responded according to their focus of attention immediately prior to when the scale was presented.

##### Physical Activity Enjoyment Scale (PACES; Kendzierski & DeCarlo, 1991)

The PACES is an 18-item measure, based on the stem statement, “Rate how you feel at the moment about the physical activity you have been doing.” Responses are provided on a 7-point Likert scale with verbal anchors (e.g., “I enjoy it” – “I hate it”). Cronbach’s α was .97, .94, and .93 for the control and experimental conditions.

##### Prefrontal Oxygenation (ΔHbDiff)

A wireless, continuous-wave fNIRS device (PortaLite, Artinis Medical Systems) was used to record oxygenated (O_2_Hb) and deoxygenated hemoglobin (HHb) in the right dorsolateral prefrontal cortex (dlPFC). The sensor was positioned at F4 according to the international 10–20 electrode placement system. Data were recorded continuously from 60 seconds prior to the warm-up until the end of the 20-minute exercise session. Haemoglobin difference (HbDiff) was calculated by subtracting HHb from O_2_Hb. HbDiff represents oxygen supply versus demand, and it is considered an index of cerebral activation (see Tempest et al., 2014). The change in the HbDiff (ΔHbDiff) was calculated by subtracting the mean HbDiff recorded during the baseline 60 seconds from the mean HbDiff recorded during the conditions.

### Procedure

#### Stage 1 (Identifying Appropriate Workloads and Familiarization)

Participants completed a graded exercise test on a cycle ergometer (Lode Corival) to identify their ventilatory threshold (T_vent_) and $\dot{V}O_{2}$ peak. Gas exchange data were collected breath-by-breath (Metalyzer) and heart rate was recorded throughout. The highest 20-s average of $\dot{V}O_{2}$ was designated as $\dot{V}O_{2}$ peak. T_vent_ was identified with the aid of software (WinBreak 3.7, Epistemic Mindworks).

The experimental setup and measures were explained to participants following the graded exercise test. Participants were familiarized with the VR headset (Oculus Rift), and they were then engaged in a short bout of very low intensity exercise on the bicycle while wearing the Oculus Rift.

#### Stage 2 (Experimental and Control Conditions)

This was a repeated measures design with a counterbalanced order of conditions. All conditions were conducted individually on a bicycle (Decathlon Elops 100 Step Over Classic Bike) mounted on a turbo trainer (Tacx Vortex) in a laboratory (see Supplementary Materials 1). All conditions comprised a 3-minute warm-up at 50% of exercise workload, followed by 20-minutes of exercise, and ended with a 2-minute cool-down at 50% of exercise workload. FS was recorded prior to warm-up, four times during exercise (at 5-, 10-, 15-, and 20-minutes), and immediately following cool-down. Attentional focus was recorded four times during exercise (at 5-, 10-, 15-, and 20-minutes). The PQ, SSQ, and PACES were completed once the participant had dismounted the bicycle. The workload during the exercise segment of the Control and 360° video condition was the external power output (Watts) at T_vent,_ as recorded during the graded exercise test. The workload during the exercise segment of the VR condition was dynamically set, based on the terrain of the virtual environment (e.g., uphill or downhill) and user input (e.g., pedal cadence). Warm-up and cool-down workloads for the VR condition were the same as for the other two conditions.

##### Control Condition

Participants exercised in a visually and auditorily sterile environment. Measures were presented by the researcher at the specified timepoints.

##### Virtual Reality Condition

A custom made virtual environment was created in Unity (https://unity.com/) to afford cycling opportunities in a pleasant environment. The virtual environment was nature-based (see Supplementary Materials 2) and permitted participants to cycle freely around the virtual mountain forest trail. A nature soundtrack was used to accompany the visuals. The landscape included gravel paths through tree-lined areas and was punctuated with hills and a lake. Participants were instructed to “maintain an intensity that you can sustain for 20 minutes” (see Parfitt et al., 2000). The Tacx Vortex turbo trainer communicated with the Unity-based virtual environment via the Ant+ (https://www.thisisant.com/) protocol. Two-way communication enabled the virtual environment to dynamically relay the simulated terrain gradient, responding to which the turbo trainer set the resistance experienced by the subject. Likewise, the turbo trainer communicated the instantaneous speed, which was used to set the speed of the bike and rider in the virtual world.

##### 360° Video Condition

Custom made high-definition footage was created using six GoPro 5 cameras mounted on a camera stabilizer (MOZA Guru 360° Air Camera Stabilizer), filmed from a tandem bicycle on a forest road near a reservoir (see Supplementary Material 3). The footage was stitched to create a 360° video and a nature soundtrack was dubbed on to the video. Participants had a 360° view throughout the video and could choose to look anywhere as the moving footage displayed a pleasant rural route. The video footage was not synchronized to bike speed, and playback speed did not alter relative to user input. The two experimental conditions were designed to have comparable immersion, but different levels of presence.

### Data Analysis

Following checks for the suitability of parametric analysis, the following analyses were conducted (a) PQ was analyzed with a paired-samples *t* test; (b) SSQ and %HRpeak were analyzed with a repeated measures ANOVA; (c) FS was analyzed with a 3 (Conditions) x 3 (Times) repeated measures ANOVA (time points were baseline, averaged in-task responses, and post task); and (d) attentional scale data, PACES, and ΔHbDiff were analyzed using separate repeated measures ANOVAs (in-task attentional scale responses were averaged). Bonferrroni corrections were applied to multiple comparisons. Statistical probability *(p*) was set at *p* < .05, and *p* values, effect sizes (*d*), and explained variance (η_p_^2^) are reported.

## Results

Data were suitable for parametric tests and violations of sphericity are reported using the conservative Greenhouse-Geisser adjustment to the degrees of freedom.

### Manipulation Checks

#### Presence Questionnaire

A paired samples *t*-test indicated that the VR condition promoted a greater sense of presence compared to the 360° video condition, *t* (11) = 3.045, *p* = .011, *d* = .91 (see Table 1).

**Table 1.** Means (and standard deviations) Statistics for Manipulation Checks and Outcome Measures.

	Control	VR	360° Video
Manipulation checks			
PQ		102.67 (12.72)	88.33 (18.17)
SSQ	19.01 (13.39)	23.69 (12.18)	18.70 (8.73)
% HRmax	78.73 (8.94)	73.38 (10.59)	79.73 (7.13)
Outcome measures			
Attention scale	38.13 (20.33)	53.92 (16.13)	56.35 (12.92)
PACES	70.83 (21.92)	88.00 (17.90)	86.58 (16.00)
ΔHbDiff (μmol/l)	9.65 (4.65)	5.20 (5.90)	7.43 (4.59)

#### Simulator Sickness Questionnaire

A repeated measures ANOVA revealed no significant main effect of Condition, *F* (2, 22) = 1.546, *p* = .235, η_p_^2^ = .12 (see Table 1).

#### Exercise Intensity (% HRmax)

A repeated measures ANOVA indicated no significant main effect of exercise Condition on exercise intensity, *F* (1.371, 15.085) = 2.859, *p* = .102, η_p_^2^ = .21 (see Table 1).

### Outcome Measures

#### Feeling Scale

**Figure 1.**
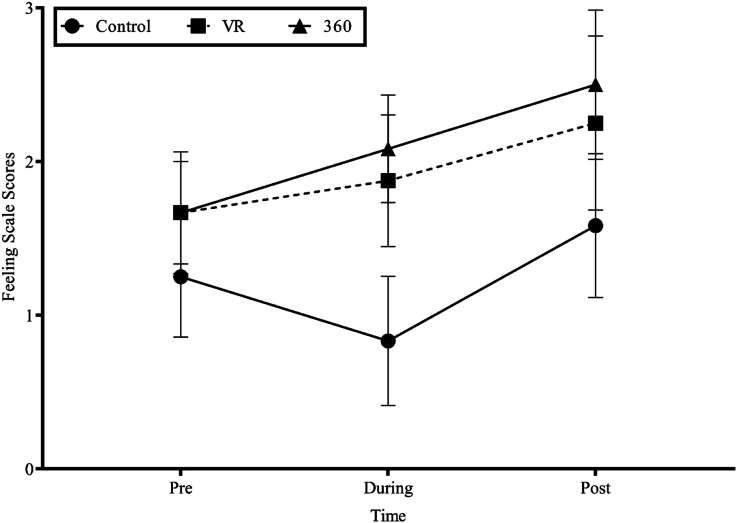
Feeling Scale Scores Before, During, and After Conditions.

A 3 × 3 repeated measures ANOVA showed a significant main effect of Condition on FS responses, *F* (2, 22) = 4.212, *p* = .028, η_p_^2^ = .28, but no main effect of Time, *F* (2, 22) = 2.383, *p* = .116, η_p_^2^ = .18, and no significant interaction effect of Condition by Time, *F* (2.53, 27.859) = .670, *p* = .553, η_p_^2^ = .06 (see Figure 1). Follow-up pairwise comparisons indicated that the 360° video condition elicited greater pleasure than the control condition (*p* = .007, *d* = .75).

#### Attentional Focus

A repeated measures ANOVA showed a significant main effect of Condition on Attentional Focus, *F* (2, 22) = 5.172, *p* = .014, η_p_^2^ = .32 (see Table 1), with follow-up pairwise comparisons indicating significantly greater dissociation during the 360° condition compared to the control condition (*p* = .023, *d* = 1.07).

#### PACES

A repeated measures ANOVA showed a significant main effect of Condition on the PACES measure of enjoyment, *F* (2, 22) = 4.254, *p* = .027, η_p_^2^ = .28 (see Table 1). Follow-up pairwise comparisons indicated that the 360° video condition was more enjoyable than the control condition (*p* = .029; *d* = .82).

#### ΔHbDiff

A repeated measures ANOVA showed a significant effect of Condition on *ΔHbDiff,* F (2, 22) = 6.057, *p* = .008, η_p_^2^ = .36 (see Table 1). Follow-up pairwise comparisons indicated a greater dlPFC activation during the control condition compared to VR (*p* = .008, *d* = .84).

## Discussion

In the present study we sought to examine whether presence in virtual environments was a significant determinant of affective responses during exercise and to explore possible neural mechanisms underpinning affective responses during exercise. Data did not support the hypothesis that a greater sense of presence elicited more positive affective responses. Further, we hypothesized that the VR condition would promote a greater sense of dissociation owing to greater presence in the virtual environment, but this was not supported.

The greatest pleasure and enjoyment were reported by participants during and following the 360° video condition. Moreover, the 360° video condition resulted in greater self-reported dissociation than did the control condition. These findings appear to support previous work showing that dissociation and affective responses to exercise are related (Jones et al., 2014). We anticipated that a greater sense of presence in the virtual environment (e.g., as a consequence of greater motor affordances during this experience) would be a more effective distractor (from bodily sensations like muscle pain) than the similarly high-immersive 360° video condition that had less interaction, owing to the participants’ greater feelings of *being there* in the virtual environment condition (Heeter, 1992)*.* However, our data indicated, instead, that a highly-immersive condition with a lower sense of presence was more distracting (and consequently pleasurable) during exercise. A possible reason for this finding is the quality of the virtual experience during our high presence condition. The Uncanny Valley, a term credited to Mori (Mori, 1970) and revisited by Mori et al., (2012), concerns human interaction with robots or virtual characters. They proposed that characters that are nearly-but-not-quite human are considered as aversive (Pan & Hamilton, 2018). Related to the present study, the VR environment might have been nearly-but-not-quite close enough to natural cycling movements, creating a slight aversion to the experience. For example, when cycling down a slight dip in the landscape, the user might have expected to feel a slight “drop” in their stomach (that did not occur), or there might have been a slight lag from the participants’ behavior of pedalling harder up a hill to the change in the virtual environment. These “nearly-but-not-quite” experiences might have created a sense of aversion as previously described (de Borst & de Gelder, 2015).

With regards to the present study, the 360° video condition might have been sufficiently distinct from “real” (e.g., with no link between pedal cadence and speed of video footage) to avoid this aversive perception. Whereas the VR condition created a greater sense of presence and resulted in a more realistic virtual experience (e.g., speeding up and slowing down to cadence), this experience may not have been sufficiently accurate to be experienced as pleasant. While participants felt “there”, “being there” may have been unnerving, owing to minor discrepancies between expected and actual outcomes. Therefore, unless a virtual environment experience (and sense of presence) can be sufficiently accurate, highly immersive experiences may be more beneficial for motivating exercise adherence.

Apart from presence, there may be other qualities of greater importance for creating an engaging virtual environment for exercise. Current options for designing virtual environments limit the extent to which environments appear “real” (physical fidelity; Kittel et al., 2020). Graphical capabilities of virtual reality are continually improving, but the photo-realism of 360° video might be of greater relevance during exercise. That is, virtual environments with high physical fidelity might be a pragmatic next step for immersive experiences during exercise, given the relative ease and cost at which they can be created, compared to environments with high psychological fidelity (the extent to which the environment replicates the perceptual-cognitive demands of the real task [Gray, 2019]). Virtual environments that can create both high psychological and physical fidelity would likely be a positive improvement on either of the experimental conditions in the present study, but they are currently difficult to achieve.

Theoretical and empirical works have described and demonstrated the salience of affective responses in determining future decisions about engaging in exercise (Brand & Ekkekakis, 2018; Rhodes & Kates, 2015; Williams et al., 2012). In the present study, we demonstrated a strategy that could be employed during exercise to facilitate a pleasant experience at a physiologically beneficial intensity. In addition to evidence supporting other extrinsic strategies (e.g., music [Karageorghis & Jones, 2014], music videos [Hutchinson et al., 2015], and imagery [Stanley & Cumming, 2010]), the immersive conditions in the present study can be added to a list of available extrinsic strategies. In line with Affective-Reflective Theory (Brand & Ekkekakis, 2018), creating pleasant exercise experiences can shape the (Type I) automatic affective valuations of exercise and lead to greater engagement with exercise owing to reduced reliance on Type II processes, and limited self-control resources. Additional empirical work examining a dose-response relationship between consistently positive exercise experiences and changes in automatic associations would be worthwhile.

### Cerebral Activation (ΔHbDiff)

In support of our hypothesis, the control condition led to the greatest activation of the dlPFC brain region. This finding offers support for the assertion that less pleasant exercise is associated with greater prefrontal cortical activation, seemingly in an attempt to cognitively mediate unpleasant affect (Jones & Ekkekakis, 2019). The least activation was recorded during the high presence (VR) condition, but these data did not entirely mirror the participants’ reports of their feelings through FS data. The increased presence in VR appeared to reduce the demand on the dlPFC compared to the Control condition, possibly indicating that the influence of aversive interoceptive sensations is minimized through activation of other brain regions not captured in the present study.

Additional examination of neural mechanisms underpinning the efficacy of these strategies is warranted. The limitations of fNIRS preclude an examination of other possible relevant brain regions (Ekkekakis, 2009). The activation of the motor cortex to plan and execute movements (of which there was a greater requirement in the VR condition) might be of relevance during high-presence VR conditions owing to the greater physical interaction with the virtual environment. Other techniques such as fMRI or electroencephalography might offer greater insights to other relevant brain regions.

Immersion appears to be a relevant consideration in understanding brain activation during exercise, as the control condition resulted in greater prefrontal cortex activation than both the highly immersive experimental conditions. Virtual stimuli were administered using the same immersive technology (Oculus Rift) and it appears that immersion in a stimulus, rather than presence, is critical to reducing prefrontal cortex activation in the regulation of feelings during exercise.

### Manipulation Checks

The similarity in HR between conditions indicates that all conditions were of comparable exercise intensity. This is an important consideration given the central role that exercise intensity plays in determining affective responses (Jones & Zenko, 2021). The statistically significant difference for responses to the PQ indicated that the experimental conditions differed as intended with greater presence in the VR condition. Responses to the SSQ indicated that experimental conditions were below a threshold considered indicative of a “bad” intervention (Caserman et al., 2021).

### Technical Considerations

The nature-based virtual environment and the 360° video were deemed suitable strategies for positively influencing affective states during exercise, owing to previous research on “green” exercise (e.g., Lahart et al., 2019). Exercise in the presence of real and digital nature has been shown to improve affective states through psychological restoration (e.g., Wooller et al., 2018), and a free-to-roam virtual nature environment provides affordances for cycling. However, other virtual environments that elicit a strong sense of presence could lead to different results (e.g., game-based environments).

Other elements of design can promote presence (e.g., haptic), and stimuli that include appeal to several senses could result in different outcomes providing the stimuli can overcome other issues (e.g., the uncanny valley [de Borst & de Gelder, 2015]). This preliminary study indicates that presence might not be of central importance during VR-based exercise and that immersion is sufficient; but further research is required to replicate this finding. The development of an interactive photorealistic virtual space would likely garner increases in pleasure during exercise, but this appears temporally distant.

While this data supports previous work with regards to immersion leading to greater pleasure (e.g., Jones & Ekkekakis, 2019), further understanding and consideration of the type of immersive content that could maximise these feelings of pleasure is required. A conceptual framework that captures the relevant factors in developing of immersive stimuli during exercise is warranted. However, a significant expansion in the empirical data available on this topic would be required before such a conceptual framework could be developed.

### Practical Implications

In addition to previous work (e.g., Bird et al., 2020; Jones & Ekkekakis, 2019), these data are of importance to professionals involved in prescribing exercise (e.g., exercise referral and cardiac rehabilitation specialists, physiotherapists). Augmenting prescribed exercise with suitable immersive stimuli is apt to help people adhere to exercise programs through greater enjoyment of the sessions. There are also implications in these data for the burgeoning commercial industry related to home and gym-based exercise equipment (e.g., Zwift, [Zwift Inc, California, USA]). Our findings offer evidence that future software development should address better facilitating pleasant experiences during exercise via improved virtual reality simulations.

### Limitations and Directions for Further Research

We created custom made stimuli to address the research questions in this study, resulting in experimental conditions that differed in the sense of presence while maintaining similar immersion. However, the development of these conditions was a time-intensive process that involved several iterations of the VR and 360° conditions prior to their experimental use, with particular care being taken to avoid such pertinent problems as cybersickness (cf. Calogiuri et al., 2018). To overcome cybersickness, the 360° video was recorded using a camera stabilised mount and processed with digital stabilisation.

Our small sample size raises the possibility of Type 2 statistical inference errors that might have led to differences between the conditions not being detected. Another consequence of the small sample was the repeated measure design that created the possibility of *halo effects* (Nisbett & Wilson, 1977) from one condition to the next, and responder fatigue owing to the number of measurements. While attempts were made to minimize these possible confounds through counterbalancing the order of conditions, future similar work might consider a sufficient sample to permit between-groups designs. A small sample size also makes generalization of these findings to larger populations suspect. Clearly, this work mut be cross validated, ideally with larger samples.

Our decision to prioritize minimizing order effects on outcome measures by counterbalancing conditions risked creating different exercise intensities between conditions. To reduce this risk, we followed Parfitt et al. (2000) by giving participants instructions to regulate their effort for the duration of the activity. Analysis of HR showed comparable workloads across conditions, as indicated by an absence of statistically significant HR differences.

## Conclusion

Cycling in a virtual green environment with a verdant landscape can improve the exercise experience. In this study, we found that 360° video footage of a forest road near a reservoir was more pleasant than an exercise condition involving a VR environment (forest trail) that created a greater sense of presence. Our data must be considered preliminary, given our small sample size and the need to have all participants experience all three research conditions, even in a counter-balanced order. While our study should be replicated with more participants, our data suggest that immersion in an emotionally positive stimulus is of greater relevance than sense of presence for fostering positive affect during exercise. Prefrontal cerebral hemodynamic responses showed reduced dlPFC activation during the VR condition compared to the control condition, indicating greater cognitive demands during the control condition, seemingly associated with regulating affect. Further research to develop innovative strategies to promote pleasant exercise experiences and perhaps more realistic virtual reality experiences are yet needed. Meanwhile, exercise prescribers and practitioners may enjoy greater adherence when they are able to immerse their exercise experience in pleasant verdant environments, perhaps even by video.

## Supplemental Material

Supplemental Material - Green and Pleasant Lands: The Affective and Cerebral Hemodynamic Effects of Presence in Virtual Environments During Exercise Click here for additional data file. Supplemental Material for Green and Pleasant Lands: The Affective and Cerebral Hemodynamic Effects of Presence in Virtual Environments During Exercise by Leighton Jones and Jonathan Wheat in Perceptual and Motor Skills
