# Supplementary material for: Green and Pleasant Lands: The Affective and Cerebral Hemodynamic Effects of Presence in Virtual Environments During Exercise
Source: Percept Mot Skills. 2022 Dec 21;130(2):826–43. doi: 10.1177/00315125221146614 (PMC10052422; doi:10.1177/00315125221146614)

Supplementary Material 1

Laboratory setup for conditions

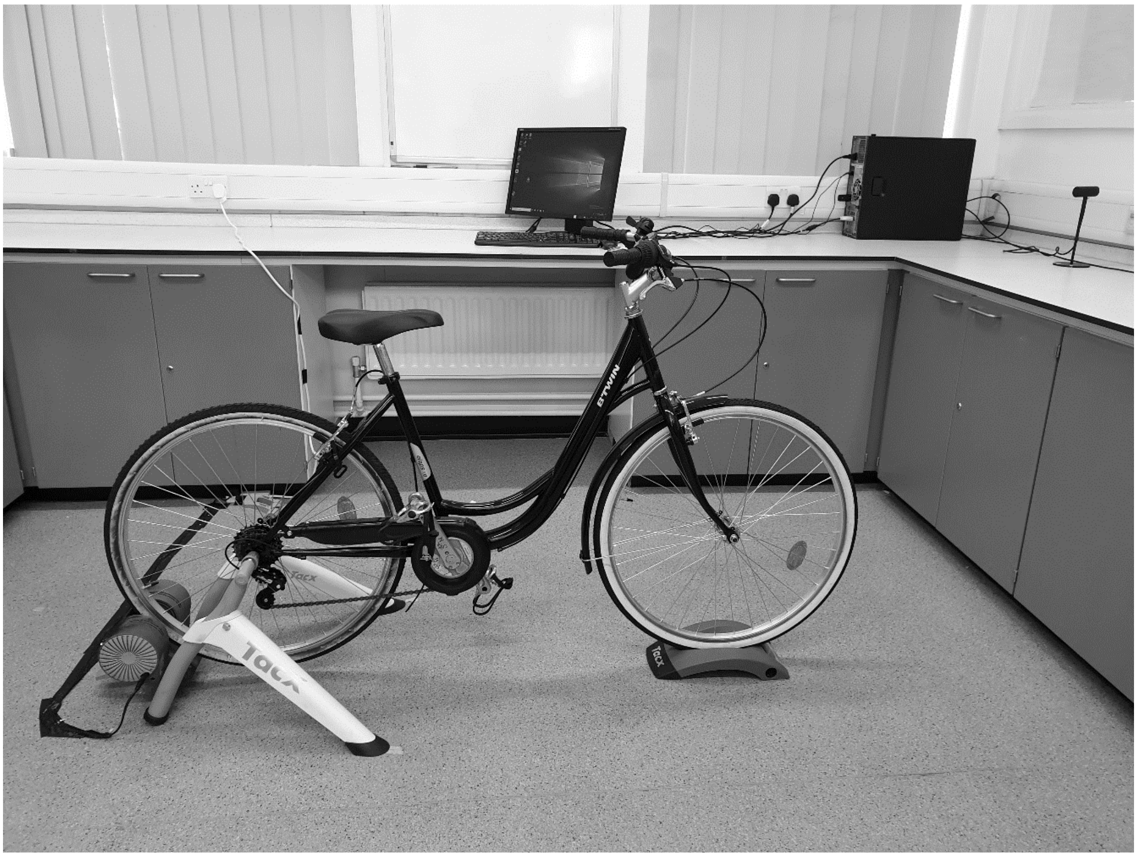

Supplementary Materials 2

Example footage for the VR condition

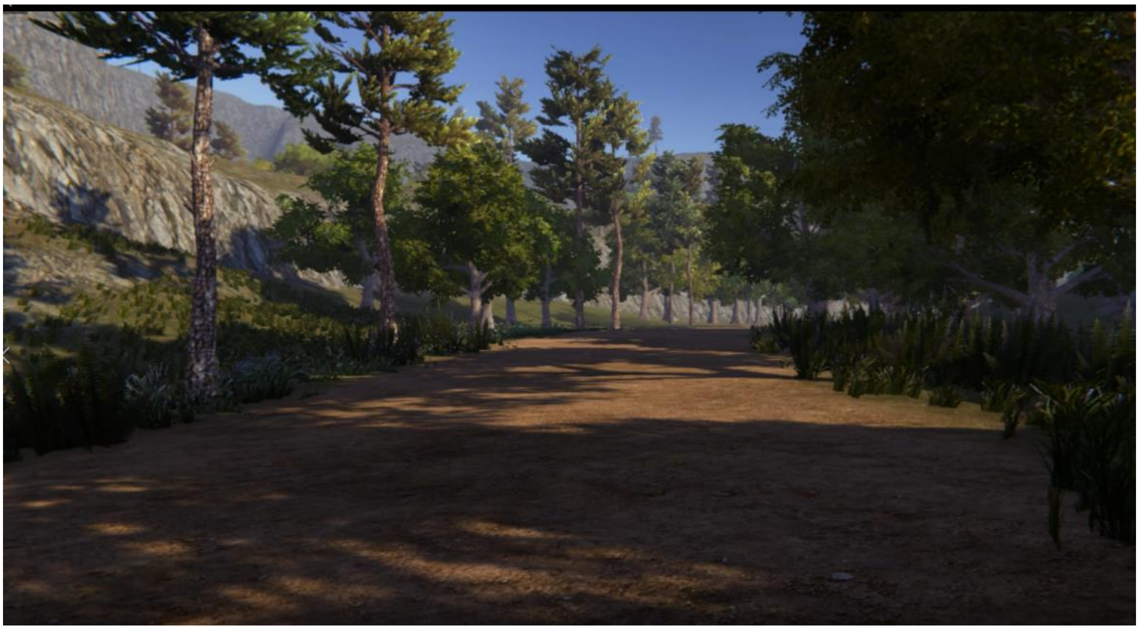

Supplementary Materials 3

Example footage of the 360° video condition

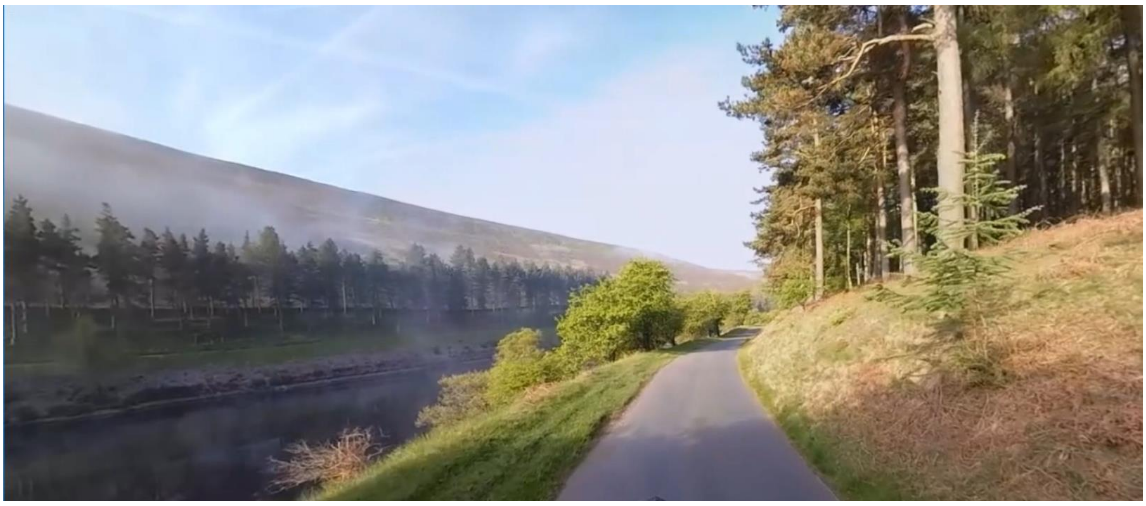

Supplement: Supplemental Material - Green and Pleasant Lands: The Affective and Cerebral Hemodynamic Effects of Presence in Virtual Environments During Exercise [file sj-pdf-1-pms-10.1177_00315125221146614.pdf]
